# Clinical research updates

**DOI:** 10.1111/camh.70036

**Published:** 2025-09-21

**Authors:** Marinos Kyriakopoulos, Athanasios Beladonas, Dimitrios Lappas, Spyridoula Kyriakopoulou

**Affiliations:** ^1^ National and Kapodistrian University of Athens Athens Greece; ^2^ Department of Child and Adolescent Psychiatry, Institute of Psychiatry Psychology and Neuroscience King's College London London UK; ^3^ South London and Maudsley NHS Foundation Trust London UK; ^4^ European Institute of Counselling & Psychotherapy Chania Greece; ^5^ Birkbeck University of London London UK

## Parental psychopathology before and after the child's diagnosis of a mental disorder

Athanasios Beladonas

European Institute of Counseling and Psychotherapy

Childhood mental disorders may have a significant impact on parental mental health. Parents of children diagnosed with mental disorders seem to be at elevated risk for depression and anxiety, which makes parenting more challenging. However, there is limited research on the range of psychopathology parents may experience before and after their child is diagnosed with a mental disorder and on the temporal association between parental and child psychopathology.

In order to investigate this relationship, Chatwin et al. (2025) conducted a population‐based matched cohort study using Danish register data. Each child diagnosed with attention‐deficit/hyperactivity disorder, autism spectrum disorder, intellectual disability, anxiety disorder, mood disorder, eating disorder, substance use disorder, and schizophrenia spectrum disorder by the age of 18 years during the period 1999–2014 and their parents were matched with 10 children of the same sex and birth year who did not have a mental health diagnosis and their parents. The yearly incidence proportion of parental mental disorders and prescriptions for psychotropic medications 4 years before and after the child's diagnosis were calculated.

The study identified that the incidence proportions of parental mental disorders in each diagnosis were relatively low, but parents of children with mental disorders had significantly higher psychopathology compared to parents of children without a mental disorder. This was higher in mothers than in fathers in both groups of children. The most common parental diagnoses were anxiety disorders and mood disorders, for which odds ratios (ORs) were, on average, double for parents of children with a diagnosis compared to those without. Peaks in the incidence of parental mood disorders were observed in the 1–2 years before and the 1–2 years after the child's diagnosis. Such peaks in parental anxiety disorders were observed 1 year after the child's diagnosis among parents of children with ADHD or ASD, and in the same year with the child in other childhood mental disorders. On average, parents of children with a diagnosis also had twice the odds of prescriptions for antidepressants and antipsychotics while parental prescriptions for ADHD medication were significantly elevated in most diagnoses, especially in parents of children with ADHD (ORs up to 17.7 for mothers and 11.9 for fathers but with relatively low yearly incidence proportions). Peaks in the incidence of parental prescriptions for sleep medication/sedatives were observed 3 years before and in the same year as the child's diagnosis. Parental antipsychotic and antidepressant prescriptions incidence peaked in the same year as the child's diagnosis while antidepressant prescriptions also peaked 1–2 years before it. Peaks in the incidence of parental prescriptions for ADHD medication were observed 1 year after the child's diagnosis among parents of children with ADHD or ASD.

Limitations of the study include not evaluating the role of genetics, which was outside the scope of it; the fact that the Danish registers only contain hospital‐based diagnoses, which may not capture children and parents with less severe psychopathology; and not fully controlling for socioeconomic status.

The pattern of the study findings may indicate that parents of children with mental disorders have a significantly increased risk of psychopathology, that the years before a child is diagnosed are particularly stressful for their parents, and that the child's diagnosis may provide some relief to their parents and even trigger additional assessment or treatment for their own mental health issues.

## Is familial expressed emotion associated with self‐harm and suicidality in childhood and adolescence?

Dimitrios Lappas

Birkbeck, University of London

Youth self‐harm and suicidality are significant public health issues. Suicide ranks among the leading causes of death for young people globally, and the incidence of self‐inflicted injuries in this population is increasing. Growing evidence indicates that familial Expressed Emotion (EE) is linked to adverse mental health outcomes in young people. EE is comprised of specific negative and positive sub‐dimensions such as criticism, emotional over‐involvement, hostility, or even warmth and positive remarks. The relationship between EE and youth self‐harm and suicidality has not been systematically evaluated.

Kennedy‐Turner et al. (2025) conducted a systematic review of the literature examining associations between EE, its specific sub‐dimensions, and self‐harm and suicidality in young people, up to the age of 24 (mean age 11.32). The current review included a total of 20 studies, 13 cross‐sectional and 7 longitudinal, carried out from 1996 to 2025, with 5752 children, adolescents, and young adults and 2690 parents and carers from seven different countries.

The researchers found that negative familial EE, especially criticism from the parents, is positively correlated with self‐harm and suicidality in the youth. It is possible that being exposed to elevated levels of parental criticism led young people to internalize the parent's critical voice, with the consequence of frequent self‐criticism, thus increasing the risk of self‐harm. Moreover, the impact of parents' criticism on their child could have a negative impact on the quality of the parent–child relationship, thus increasing alienation of the parent and a thwarted sense of belonging, both of which were identified as mediators. Self‐criticism was also identified as a significant mediator. Gender was found to be a moderator, as both the caregiver's and young person's gender could influence the strength of associations with psychopathology. Total negative paternal EE significantly predicted higher suicidal ideation and re‐attempt, in contrast to total negative maternal EE. Finally, in the case of non‐suicidal self‐injury, depressive symptoms, attachment anxiety, and difficulties with reflective functioning could act as mediators in the relationship with EE. This suggests that EE may contribute to self‐harm and suicidality indirectly by shaping insecure attachment patterns and unhelpful ways of thinking linked to mental health struggles.

The review indicated that EE could also be a risk factor for youth self‐harm and suicidality alongside negative family environmental factors such as familial conflicts, stress, dysfunctional communication, and disrupted attachment. It remains unclear whether EE is a cause or an outcome of youth psychopathology, as it may represent a transactional, bidirectional interaction that reflects relational communication challenges within families, rather than a fixed parental trait.

Although the large sample size of this review as well as the fact that it delves into an unexplored gap in literature constitute strengths of this study, there are significant limitations which must also be considered. The majority of the studies examined in this review were cross‐sectional, which limits the ability to draw conclusions regarding causality. Furthermore, almost all included studies were conducted in Western countries, which affects sample diversity and possibly introduces cultural bias. Finally, there was variability in how EE was assessed across studies, which may affect the comparability of their findings.



Kennedy‐Turner, J.
, 
Nemeth, Z.
, & 
Anderson, L.
 (2025). Is familial expressed emotion associated with self‐harm and suicidality in childhood and adolescence? A systematic review. Adolescent Research Review. 10.1007/s40894-025-00261-7


## Student‐ and school‐level factors associated with mental health and well‐being in early adolescence

Spyridoula Kyriakopoulou

National and Kapodistrian University of Athens

The experience young people have in educational settings may have a significant influence on their mental health and well‐being. The extent of this influence may be related to student characteristics and school contextual factors, including its community composition, operational features, sociocultural aspects, and adverse exposures such as bullying. Identifying the relative importance of these factors could inform the design of targeted school‐based interventions to promote student resilience.

Hinze et al. (2024) explored this using longitudinal data of 8,376 students (55% female; aged 11–14 years at baseline) from a representative sample of 84 schools in the United Kingdom. Data were collected as part of a cluster randomized controlled trial over two academic years, with follow‐up at 1, 1.5, and 2 years. The authors assessed risk for depression using the Center for Epidemiologic Studies Depression Scale, social‐emotional‐behavioral difficulties with the Strengths and Difficulties Questionnaire, and well‐being with the Warwick‐Edinburgh Mental Well‐Being Scale. They also evaluated the relationships of these measures with student‐ and school‐level factors, including various indicators and the Alaska School Climate and Connectedness Survey, through multilevel regression models.

The study identified that students' mental health and well‐being were within normal ranges at initial assessment, but depression risk scores fell into the at‐risk range at follow‐up. This was more pronounced in girls, with older age being significantly associated with worse mental health over time. Inter‐school differences accounted for a small yet statistically significant proportion of the variance in young people's mental health difficulties and well‐being. Student‐rated school climate – encompassing the experience of feeling safe, connected, and welcome – as a time‐varying factor both at the student‐ and the school‐level was identified as the factor most strongly related to the positive mental health and well‐being of adolescents.

Strengths of this study include the representativeness and size of its sample and the use of validated measures and rigorous analyses. The exclusion of schools lacking an adequate special educational needs strategy, the relatively short follow‐up periods, some missing data, and the non‐inclusion of potentially relevant variables which were outside the scope of this study are some of its limitations.

The finding that school climate may be a significant factor in adolescent mental health, beyond other contextual, community, or operational factors, has important implications for the design of policies and system‐level interventions aimed at its improvement.



Hinze, V.
, 
Montero‐Marin, J.
, 
Blakemore, S.J.
, 
Byford, S.
, 
Dalgleish, T.
, 
Degli Esposti, M.
, … & 
Kuyken, W.
 (2024). Student‐ and school‐level factors associated with mental health and well‐being in early adolescence. Journal of the American Academy of Child and Adolescent Psychiatry, 63, 266–282.37866473
10.1016/j.jaac.2023.10.004PMC10935542

## Conflict of interest statement

M.K. is the *CAMH* Associate Editor for Clinical Research Updates. The editor thanks the contributors for this issue's Clinical Research Updates. The editor has declared that he has no competing or potential conflicts of interest.

## Ethics statement

No ethical approval was required for these updates.

## Data Availability

Data sharing is not applicable to this article as no new data were created or analyzed.
